# Reinitiation and Subsequent Discontinuation of Antiplatelet Treatment in Nonpersistent Older Patients with Peripheral Arterial Disease

**DOI:** 10.3390/biomedicines9091280

**Published:** 2021-09-21

**Authors:** Martin Wawruch, Jan Murin, Tomas Tesar, Martina Paduchova, Miriam Petrova, Denisa Celovska, Beata Havelkova, Michal Trnka, Emma Aarnio

**Affiliations:** 1 Institute of Pharmacology and Clinical Pharmacology, Faculty of Medicine, Comenius University, 811 08 Bratislava, Slovakia; miriampetrova1@gmail.com; 2 1st Department of Internal Medicine, Faculty of Medicine, Comenius University, 813 69 Bratislava, Slovakia; jan.murin@gmail.com (J.M.); denisa.celovska@gmail.com (D.C.); 3 Department of Organisation and Management of Pharmacy, Faculty of Pharmacy, Comenius University, 832 32 Bratislava, Slovakia; 4 Department of Angiology, Health Centre, 917 01 Trnava, Slovakia; martina.paduchova@medena.sk; 5 General Health Insurance Company, 851 04 Bratislava, Slovakia; beata.havelkova@vszp.sk; 6 Institute of Medical Physics, Biophysics, Informatics and Telemedicine, Faculty of Medicine, Comenius University, 813 72 Bratislava, Slovakia; michal.trnka@fmed.uniba.sk; 7 Institute of Biomedicine, University of Turku, 20014 Turku, Finland; emma.aarnio@uef.fi; 8 School of Pharmacy, University of Eastern Finland, 70211 Kuopio, Finland

**Keywords:** peripheral arterial disease, antiplatelet, reinitiation, discontinuation, non-persistence, diabetes mellitus, atrial fibrillation

## Abstract

The successful treatment of peripheral arterial disease (PAD) depends on adequate adherence to medications including antiplatelet agents. The aims of this study were (a) to identify the proportion of nonpersistent patients who reinitiated antiplatelet therapy and how many of them discontinued therapy after reinitiation, and (b) to identify patient- and medication-related characteristics associated with the likelihood of reinitiation and discontinuation among reinitiators. The analysis of reinitiation was conducted on 3032 nonpersistent users of antiplatelet agents aged ≥65 years, with PAD newly diagnosed in 2012. Discontinuation (i.e., a treatment gap of ≥6 months without antiplatelet medication prescription) was analysed in 2006 reinitiating patients. To identify factors associated with the likelihood of reinitiation and discontinuation, Cox regression with time-dependent covariates was used. Reinitiation was recorded in 2006 (66.2%) of 3032 patients who had discontinued antiplatelet medication. Among these 2006 reinitiators, 1078 (53.7%) patients discontinued antiplatelet therapy again. Ischemic stroke and myocardial infarction during nonpersistence and bronchial asthma/chronic obstructive pulmonary disease were associated with an increased likelihood of reinitiation. University education was associated with discontinuation among reinitiators. Factors associated with the probability of reinitiation and discontinuation in reinitiators make it possible to identify older PAD patients in whom “stop-starting” behaviour may be expected.

## 1. Introduction

Peripheral arterial disease (PAD) represents a manifestation of systemic atherosclerosis. In our manuscript, PAD refers to atherosclerotic process affecting arteries of the lower limbs. It is estimated that PAD affects 10–15% of the general population. The prevalence of PAD increases with advancing age, reaching more than 20% among persons aged >80 years [1,2,3]. Since atherosclerosis is a generalised process that affects the whole vasculature, PAD is associated with a 6-fold increase in the risk of major adverse cardiovascular (CV) events (nonfatal myocardial infarction (MI), nonfatal stroke, and CV death). PAD is associated with an annual mortality rate of 4–6% [4,5,6,7]. The clinical presentation of the disease may vary from asymptomatic disease to intermittent claudication, atypical leg pain, rest pain, ischemic ulcers, or gangrene, which often requires limb amputation. Approximately 50% of PAD patients have no symptoms. This is associated with underdiagnosis and undertreatment of the disease [1,8]. The disease can progress to critical limb ischemia, which represents the end stage of PAD and is associated with a high rate of limb loss and patient mortality. Risk factors of PAD include the common risk factors of atherosclerosis, e.g., age, smoking, arterial hypertension, dyslipidaemia, and diabetes mellitus [5,9]. Management of PAD includes, in addition to the treatment of modifiable risk factors, administration of antiplatelet agents, statins, and inhibitors of angiotensin-converting enzyme/angiotensin receptor blockers [10,11]. PAD patients manifest platelet hyperaggregability and increased platelet activation. This underlines a key role of antiplatelet medication in the treatment of PAD [7].

The successful treatment of PAD depends on adequate adherence to the medications mentioned above. The reduction of the risk of CV and limb events as well as a decreased risk of progression of the disease to critical limb ischemia represent success in the treatment of PAD. Adherence includes three interrelated phases: initiation, implementation, and persistence [12,13]. In the literature, there are almost no studies focused on nonpersistence with antiplatelet treatment in older PAD patients. Qvist et al. [14] analysed adherence to antiplatelet and statin treatment among patients aged 65–74 years with abdominal aortic aneurysm or PAD. However, factors associated with nonpersistence were not found and the issue of reinitiation was not addressed in that study. In our previous study on nonpersistence with antiplatelet medications in older PAD patients (≥65 years), 33.0% of patients were identified as nonpersistent [15]. The following patient- and medication-related characteristics were associated with a decreased likelihood of nonpersistence: age, history of ischemic stroke or MI, clopidogrel or combination of aspirin and clopidogrel as the initial antiplatelet medication, higher patient co-payment, general practitioner as index prescriber, and a higher overall number of medications. On the other hand, female sex, atrial fibrillation, anxiety disorders, bronchial asthma/chronic obstructive pulmonary disease (COPD), being a new antiplatelet medication user in whom antiplatelet treatment was initiated in association with the diagnosis of PAD, and the use of anticoagulants or antiarrhythmic agents were associated with an increased probability of nonpersistence.

The issue of reinitiation and subsequent discontinuation among reinitiators has been rarely studied [16,17]. In addition, there are no studies in the literature focused on reinitiation and subsequent discontinuation among reinitiators of antiplatelet treatment in nonpersistent older PAD patients. For the reasons mentioned above, we carried out the study presented in this manuscript. The study included two separate analyses: (1) analysis of reinitiation among patients who became nonpersistent with antiplatelet treatment in our previous study [15], and (2) analysis of discontinuation (nonpersistence) among patients who reinitiated antiplatelet treatment. The aims of these analyses were (a) to identify the proportion of nonpersistent patients who reinitiated antiplatelet therapy and the proportion of reinitiators who discontinued this therapy again after a certain period of persistence, and (b) to identify patient- and medication-related characteristics associated with the likelihood of reinitiation and discontinuation among reinitiators.

## 2. Materials and Methods

### 2.1. Database and Study Population

Previously, we carried out a register-based retrospective cohort study which analysed nonpersistence with antiplatelet medications in older PAD patients [15]. The study cohort consisted of patients aged ≥65 years, with PAD newly diagnosed in 2012, who were administered antiplatelet therapy (*n* = 9178). At the end of the 5-year follow-up period, nonpersistence was identified on the basis of a treatment gap of at least 6 months without antiplatelet medication prescription in 3032 (33.0%) patients. The analysis of reinitiation was conducted on these 3032 patients. The selection of the whole cohort (persistent and nonpersistent patients) used in our previous study (*n* = 9178) is described in detail in the previous manuscript. The database of the General Health Insurance Company in Slovakia represented the source of data. The diagnosis of symptomatic PAD was based on an ankle–brachial index of less than 0.9, positive findings on ultrasonography of the arterial system of lower limbs, and presence of PAD symptoms (e.g., claudication, rest pain, ischemic ulcers) [10].

Two thousand and six (66.2%) of the 3032 nonpersistent patients reinitiated antiplatelet treatment during the follow-up. This sample of reinitiators represents the study cohort in our analysis of discontinuation among reinitiators.

### 2.2. Analysis of Reinitiation and Subsequent Discontinuation after Reinitiation

The index date of the analysis of reinitiation was the date of the first day of the period of nonpersistence among the 3032 patients who discontinued antiplatelet treatment in our previous study [15]. Reinitiation was defined as the first prescription of antiplatelet medication after nonpersistence (after the treatment gap). Patients were followed from discontinuation until (a) reinitiation, (b) the end of the 5-year follow-up period of our previous study, which started with the first prescription of antiplatelet agent after the diagnosis of PAD [15], or (c) death.

The index date of the analysis of discontinuation among reinitiators was the date when a patient reinitiated antiplatelet therapy. Discontinuation (nonpersistence) was defined similarly to our previous study (i.e., a treatment gap of at least 6 months without a prescription of antiplatelet medication which occurred after reinitiation) [15]. Patients in whom a treatment gap was found were classified as discontinuers (nonpersistent), and those with no such gap were considered persistent. Patients were followed from reinitiation until (a) discontinuation, (b) the end of the 5-year follow-up period of our previous study [15], or (c) death.

### 2.3. Factors Associated with Reinitiation and Discontinuation among Reinitiators

The same sociodemographic characteristics, comorbid conditions, and CV co-medication as those evaluated in our previous study [15] were analysed as factors potentially associated with the probability of reinitiation/discontinuation among reinitiators. Data on these factors were collected at the time of inclusion of patients in our previous study [15]. History of CV events (ischemic stroke, transient ischemic attack (TIA), and MI) covers events which occurred within a 5-year period before the index date of the analysis of reinitiation/discontinuation among reinitiators. Besides the history of CV events, events which occurred during the periods of nonpersistence or reinitiation were also assessed as factors potentially associated with the probability of reinitiation or discontinuation among reinitiators. In the analysis of reinitiation, the last antiplatelet agents administered before the discontinuation (before the start of the treatment gap) in our previous study [15], and the patient’s co-payment for these agents were assessed as medication-related factors potentially associated with reinitiation. In the analysis of discontinuation among reinitiators, the antiplatelet agents initially prescribed at the time of reinitiation and the patient’s co-payment for these agents were evaluated as factors potentially associated with discontinuation among reinitiators. The duration of the persistence period recorded in our previous study [15] was evaluated as a factor potentially associated with the probability of reinitiation. The duration of the period of nonpersistence after discontinuation in our previous study [15] (i.e., after the first discontinuation) was assessed as a factor potentially associated with the likelihood of discontinuation among reinitiators.

### 2.4. Statistical Analysis

Categorical variables were expressed as frequencies and percentages and continuous variables as means ± standard deviations.

Continuous variables were compared between the two patient groups using the Mann–Whitney U test. This nonparametric test was applied because of the non-Gaussian distribution of continuous variables. Normality of distribution was assessed using the Kolmogorov–Smirnov test. The χ^2^ test was applied to compare categorical variables between the two patient groups.

To illustrate how the probabilities of reinitiation and discontinuation among reinitiators changed over time, the Kaplan–Meier model was used. To identify the factors associated with the likelihood of reinitiation and discontinuation among reinitiators, Cox regression with time-dependent covariates was used. CV events which occurred during the periods of nonpersistence after discontinuation in our previous study [15] and reinitiation represented time-dependent covariates. All other patient- and medication-related characteristics included in the model were time-independent variables. The hazard ratios and corresponding 95% confidence intervals were calculated for each factor [18].

All tests were carried out at the level of statistical significance α = 0.05. IBM SPSS for Windows, version 27 (IBM SPSS Inc., Armonk, NY, USA), was used for our analyses.

## 3. Results

Reinitiation was recorded in 2006 (66.2%) of 3032 patients who discontinued antiplatelet medication after a PAD diagnosis in 2012 in our previous study [15]. Among these reinitiators, 1078 (53.7%) patients discontinued antiplatelet treatment again. The baseline characteristics of the cohorts used in the analysis of reinitiation and discontinuation among reinitiators are shown in Table 1. The mean duration of treatment after reinitiation among the patients who subsequently discontinued therapy was 7.9 ± 7.8 months.

**Figure 1:**
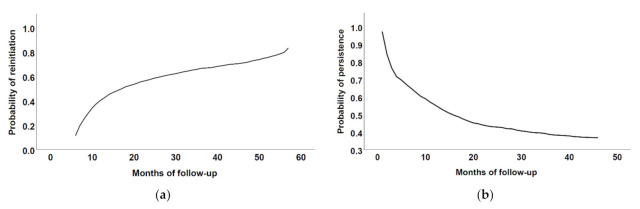
Kaplan–Meier curves of (**a**) reinitiation of antiplatelet treatment and (**b**) persistence among reinitiators during the follow-up.

The Kaplan–Meier figures show how the probabilities of reinitiation (Figure 1a) and discontinuation among reinitiators (Figure 1b) changed over time.

In the analysis of reinitiation, ischemic stroke and MI during nonpersistence and bronchial asthma/COPD appeared as factors associated with an increased likelihood of reinitiation of antiplatelet treatment (Table 2). On the other hand, history of ischemic stroke, atrial fibrillation, administration of clopidogrel or dual antiplatelet treatment (DAPT, combination of aspirin and clopidogrel) before discontinuation in our previous study [15], administration of cardiac glycosides or beta-blockers, and longer duration of persistence period before discontinuation in our previous study represented factors associated with a lower probability of reinitiation of antiplatelet therapy.

In the analysis of discontinuation among reinitiators of antiplatelet treatment, university education was associated with an increased likelihood of discontinuation, whereas increasing age, history of ischemic stroke, diabetes mellitus, and higher patient co-payment for the antiplatelet agent used initially at the time of reinitiation were associated with a decreased probability of discontinuation of antiplatelet treatment after reinitiation (Table 2).

## 4. Discussion

In our previous study, 33.0% of 9178 older PAD patients became nonpersistent with antiplatelet medication [15]. More than half (66.2%) of these 3032 nonpersistent patients reinitiated antiplatelet therapy during the follow-up period. However, more than half (53.7%) of 2006 reinitiators discontinued antiplatelet treatment again. These results suggest that reinitiation does not necessarily mean that the issue of nonpersistence has been resolved. Our results indicate a relatively common “stop-starting” behaviour which was previously reported by Vinogradova et al. [19] in their prospective open cohort study focused on discontinuation and restarting of statin therapy.

In the study by Ofori-Asenso et al. [17] focused on the analysis of switching, discontinuation, and reinitiation of statin treatment among older patients, 63.6% of 49,380 patients discontinued the treatment; subsequently, 60.4% of them reinitiated it. Among these 18,977 reinitiators, 67.6% discontinued statin treatment again. In a cohort study by Horsburgh et al. [16] which evaluated patterns of discontinuation and reinitiation in new users of metformin monotherapy, discontinuation after 1, 2, and 5 years of follow-up was reported in 28%, 37%, and 46% of patients, respectively. Reinitiation within 1, 2, and 5 years of the first discontinuation was reported in 23%, 49%, and 73% of patients, respectively. Discontinuation after the first reinitiation was also reported to be common (48% after one year). According to a cohort study focused on the analysis of discontinuation, reinitiation, and persistence patterns of statin treatment among Medicare beneficiaries after MI conducted by Booth et al. [20], in the 182 days after MI hospital discharge, 15.4% of beneficiaries discontinued statin treatment. Of this group, 53.7% reinitiated statin therapy. In the 182 days after reinitiation, only 45.8% had high adherence defined according to the proportion of days covered being ≥80%.

Higher age was associated with a decreased likelihood of discontinuation among reinitiators in our study. A similar result was found also in our previous study [15]. These results may indicate careful taking of medications in older patients. On the other hand, according to the study by Ofori-Asenso et al. [17], patients aged ≥75 years were more likely to discontinue statin therapy in comparison with those aged 65 to 74 years. In the study by Vinogradova et al. [19], older patients (≥75 years) had a higher likelihood of discontinuation and a lower probability of reinitiation of statin treatment. In that study, 41% (*n* = 57,791) of 139,314 patients with statin treatment prescribed in secondary prevention discontinued, and 75% of those who discontinued reinitiated the treatment again.

In our study, ischemic stroke and MI which occurred during the period of nonpersistence were associated with reinitiation of antiplatelet treatment. This result indicates the significant influence of acute conditions, the treatment of which also requires antiplatelet medication administration, on taking antiplatelet medication [21,22,23]. Incident MI and other CV-disease-related hospitalisations appeared to be strong predictors of reinitiation of statin treatment in the study of the dynamics of statin use by Brookhart et al. [24]. However, TIA did not appear as a factor associated with the likelihood of reinitiation in our study. The transient nature of neurological symptoms which disappear within one hour may serve as a possible explanation of insufficient awareness of the importance of antiplatelet treatment in the case of TIA patients [25]. On the other hand, history of ischemic stroke before the index date of the analyses of reinitiation and discontinuation among reinitiators was associated with a lower likelihood of both reinitiation and discontinuation among reinitiators. History of ischemic stroke was associated with persistence in our previous study as well [15]. These results indicate that patients with a history of ischemic stroke had a lower tendency to discontinue, but if discontinued then they were less likely to reinitiate, and if reinitiated then they were more likely to be persistent with the treatment.

University education appeared as a factor associated with discontinuation among reinitiators. The design of our study does not make it possible to explain this result. Better long-term refill persistency with beta-blockers was reported in patients with lower compared to higher education in the study by Rasmussen et al. [26]. However, the effect of education was significant only in the subgroup of younger patients (30–64 years).

Among co-morbid conditions, atrial fibrillation was associated with an increased probability of nonpersistence with antiplatelet medication in our previous study [15] and with a lower likelihood of reinitiation in the present study. These results may be associated with the use of anticoagulants in patients with atrial fibrillation and physicians’ awareness of an increased risk of bleeding in the case of combination of anticoagulants with antiplatelet medications [27]. Bronchial asthma/COPD was associated with an increased likelihood of reinitiation of antiplatelet treatment in this study and an increased probability of nonpersistence in our previous study [15]. These results indicate that PAD patients with bronchial asthma/COPD tend to discontinue antiplatelet treatment, but after discontinuation they are more likely to reinitiate. A higher rate of COPD was reported in patients who interrupted DAPT at least temporarily in a study by Ferreira-González et al. [28] focused on the analysis of risk of major cardiac events related to discontinuation of DAPT in patients after drug-eluting stent implantation. However, reinitiation of antiplatelet treatment was not evaluated in that study.

Diabetes mellitus was associated with a decreased probability of discontinuation among reinitiators in this study. This result may indicate a good awareness of the importance of antiplatelet medication administration in secondary prevention of PAD in patients with concomitant diabetes mellitus. According to the study by Vinogradova et al. [19], patients with type 2 diabetes mellitus were less likely to discontinue and more likely to reinitiate the statin treatment. On the other hand, in the study by Ofori-Asenso et al. [17], diabetes mellitus was associated with a higher probability of both discontinuation and reinitiation.

Among CV co-medications, administration of beta-blockers and cardiac glycosides was associated with a lower likelihood of reinitiation in our study. The design of our study does not make it possible to explain the reasons for this finding. However, besides other conditions, these two drug classes are used in the treatment of atrial fibrillation. Physicians’ awareness of an increased risk of bleeding when combining anticoagulants with antiplatelet agents in PAD patients with atrial fibrillation, as mentioned above, may serve as a possible explanation of this association [27]. On the other hand, according to the study by Ofori-Asenso et al. [17], CV pharmacotherapy including beta-blockers, antiplatelet agents, and ACE (angiotensin-converting enzyme) inhibitors or ARBs (angiotensin receptor blockers) was associated with a lower probability of discontinuation, and no association with reinitiation was reported.

Patients in whom clopidogrel or DAPT (aspirin and clopidogrel) was the last antiplatelet medication prescribed before the first discontinuation in our previous study [15] had a lower probability of reinitiating antiplatelet treatment in comparison with those treated with aspirin alone. In our previous study [15], patients in whom clopidogrel or DAPT was administered at the time of PAD diagnosis had better persistence in comparison with those treated with aspirin alone. The lower likelihood of reinitiating antiplatelet treatment in patients treated with DAPT may be related to its use after percutaneous coronary intervention in MI patients [21]. These patients seem to take DAPT during the necessary period, but they are not sufficiently aware of the necessity of continuation of antiplatelet monotherapy in secondary prevention of both MI and PAD after stopping DAPT.

Patient co-payment represented a factor associated with a lower likelihood of discontinuation among reinitiators in our study. A higher co-payment was associated with persistence in our previous study as well [15]. On the other hand, in a population-based cohort study focused on persistence with and adherence to oral antidiabetics by Simard et al. [29], the probability of nonpersistence was higher when drug co-payments were required. In a study by Mefford et al. [30], 15% of 7216 patients reported statin discontinuation. In the group of statin discontinuers, cost was reported as a potential reason for discontinuation in 3% of participants. Patients who discontinued statin treatment due to cost reported more willingness to restart therapy. However, in our study, patient co-payment was not associated with the probability of reinitiation.

Longer duration of persistence period which preceded the first discontinuation in our previous study [15] was associated with a decreased likelihood of reinitiation in the present study. This result may indicate that patients consider the antiplatelet treatment to be unnecessary after a longer period of persistence. Therefore, the physician’s explanation of the importance of lifelong administration of antiplatelet agents in PAD patients is of particular importance. In contrast to our results, Alfian et al. [31] reported a longer duration of persistence to be a predictor of reinitiation. Their study was focused on the analysis of pharmacy-based predictors of nonadherence, nonpersistence, and reinitiation of antihypertensive drugs among patients on oral diabetes drugs.

Our study has some limitations which should be taken into consideration when interpreting its findings. The database of the General Health Insurance Company, which served as a source of data for our study, was primarily developed for insurance purposes and not for research. This database does not make it possible to ascertain who decided to reinitiate or discontinue the treatment, i.e., the physician or the patient. It is also impossible to ascertain whether patients took their medications as prescribed. On the other hand, the large sample size covering all administrative regions of the Slovak Republic and precise data on patients’ co-morbid conditions and medications represent strengths of our study.

## 5. Conclusions

Our study revealed a high proportion of reinitiators among PAD patients nonpersistent with antiplatelet medication, but also a high proportion of reinitiators who discontinued treatment again. Factors associated with the probability of reinitiation and discontinuation in reinitiators make it possible to identify older PAD patients in whom “stop-starting” behaviour may be expected. In this group of patients, special attention should be paid to explanation of the importance of life-long administration of antiplatelet medications if the secondary preventive measures are to be successful. Since the beneficial effects of antiplatelet medications accrue over time, it is necessary to focus educational efforts even on patients who are likely to reinitiate in order to prevent treatment interruptions.

## Figures and Tables

**Table 1:** Baseline characteristics of the study cohorts.

Factor	Analysis of Reinitiation (*n* = 3032)			Analysis of Discontinuation Among Reinitiators (*n* = 2006)		
	Without Reinitiation (*n* = 1026)	With Reinitiation (*n* = 2006)	*p*	Persistent (*n* = 928)	Non-Persistent (*n* = 1078)	*p*
*Socio-demographic characteristics*						
Age	74.4 ± 6.0	73.3 ± 6.0	<0.001 *	73.5 ± 6.1	73.1 ± 5.9	0.231 *
Female sex	617 (60.1)	1255 (62.6)	0.193	567 (61.1)	688 (63.8)	0.209
University education	84 (8.2)	160 (8.0)	0.840	63 (6.8)	97 (9.0)	0.069
Employed patients	55 (5.4)	135 (6.7)	0.141	67 (7.2)	68 (6.3)	0.416
*History of cardiovascular events ^a^*						
History of ischemic stroke	334 (32.6)	367 (18.3)	**<0.001**	266 (28.7)	220 (20.4)	**<0.001**
History of TIA	136 (13.3)	207 (10.3)	**0.016**	137 (14.8)	127 (11.8)	**0.049**
History of MI	136 (13.3)	127 (6.3)	**<0.001**	97 (10.5)	95 (8.8)	0.213
*Cardiovascular events during non-persistence/period of reinitiation*						
Ischemic stroke	88 (8.6)	119 (5.9)	**0.006**	102 (11.0)	40 (3.7)	**<0.001**
TIA	36 (3.5)	57 (2.8)	0.313	39 (4.2)	20 (1.9)	0.002
MI	38 (3.7)	65 (3.2)	0.505	55 (5.9)	24 (2.2)	<0.001
*Comorbid conditions*						
Number of comorbid conditions	2.8 ± 1.6	2.6 ± 1.5	<0.001 *	2.5 ± 1.5	2.6 ± 1.5	0.177 *
Arterial hypertension	835 (81.4)	1498 (74.7)	**<0.001**	697 (75.1)	801 (74.3)	0.680
Chronic heart failure	85 (8.3)	91 (4.5)	**<0.001**	30 (3.2)	61 (5.7)	**0.009**
Atrial fibrillation	219 (21.3)	169 (8.4)	**<0.001**	70 (7.5)	99 (9.2)	0.187
Diabetes mellitus	397 (38.7)	730 (36.4)	0.214	367 (39.5)	363 (33.7)	**0.006**
Hypercholesterolemia	398 (38.8)	818 (40.8)	0.291	366 (39.4)	452 (41.9)	0.258
Dementia	67 (6.5)	104 (5.2)	0.129	51 (5.5)	53 (4.9)	0.560
Depression	122 (11.9)	215 (10.7)	0.331	93 (10.0)	122 (11.3)	0.350
Anxiety disorders	318 (31.0)	622 (31.0)	0.994	271 (29.2)	351 (32.6)	0.105
Parkinson’s disease	47 (4.6)	71 (3.5)	0.161	36 (3.9)	35 (3.2)	0.445
Epilepsy	21 (2.0)	46 (2.3)	0.662	24 (2.6)	22 (2.0)	0.416
Bronchial asthma/COPD	222 (21.6)	487 (24.3)	0.104	211 (22.7)	276 (25.6)	0.136
*Antiplatelet agent related characteristics*						
Antiplatelet agent ^b^						
Aspirin	672 (65.5)	1596 (79.6)	**<0.001**	697 (75.2)	848 (78.7)	**<0.001**
Clopidogrel	307 (29.9)	376 (18.7)		149 (16.1)	154 (14.3)	
Ticlopidine				25 (2.7)	45 (4.2)	
Aspirin + Clopidogrel	47 (4.6)	34 (1.7)		56 (6.0)	31 (2.9)	
Patient’s co-payment (EUR) ^c^	1.5 ± 1.0	1.2 ± 0.9	<0.001 *	1.4 ± 1.0	1.3 ± 0.9	<0.001 *
New antiplatelet agent user ^d^	146 (14.2)	431 (21.5)	**<0.001**	183 (19.7)	248 (23.0)	0.074
General practitioner as index prescriber	712 (69.4)	1340 (66.8)	0.148	642 (69.2)	698 (64.7)	**0.036**
*Cardiovascular co-medication*						
Number of medications	8.2 ± 2.6	7.5 ± 2.9	<0.001 *	7.6 ± 2.8	7.5 ± 2.9	0.514 *
Number of CV medications	5.2 ± 2.4	4.5 ± 2.1	<0.001 *	4.5 ± 2.1	4.6 ± 2.2	0.727
Anticoagulants	256 (25.0)	361 (18.0)	**<0.001**	155 (16.7)	206 (19.1)	0.162
Cardiac glycosides	95 (9.3)	71 (3.5)	**<0.001**	32 (3.4)	39 (3.6)	0.838
Antiarrhythmic agents	127 (12.4)	111 (5.5)	**<0.001**	41 (4.4)	70 (6.5)	**0.043**
Beta-blockers	214 (20.9)	334 (16.7)	**0.004**	145 (15.6)	189 (17.5)	0.253
Thiazide diuretics	248 (24.2)	430 (21.4)	0.087	204 (22.0)	226 (21.0)	0.580
Loop diuretics	232 (22.6)	290 (14.5)	**<0.001**	125 (13.5)	165 (15.3)	0.244
Mineralocorticoid receptor antagonists	79 (7.7)	68 (3.4)	**<0.001**	35 (3.8)	33 (3.1)	0.381
Calcium channel blockers	313 (30.5)	625 (31.2)	0.714	299 (32.2)	326 (30.2)	0.340
RAAS inhibitors	864 (84.2)	1607 (80.1)	**0.006**	739 (79.6)	868 (80.5)	0.620
Statin	754 (73.5)	1397 (69.6)	**0.027**	656 (70.7)	741 (68.7)	0.343
Lipid lowering agents other than statins ^e^	100 (9.7)	206 (10.3)	0.651	96 (10.3)	110 (10.2)	0.918
						
Duration of persistence/non-persistence (months) ^f^	26.4 ± 15.7	13.9 ± 12.8	<0.001 *	16.7 ± 13.5	13.6 ± 8.9	0.052 *

In the case of categorical variables, values represent the frequency and the percentages are provided in parentheses (% of *n*). In the case of continuous variables, means ± standard deviations are provided. TIA—transient ischemic attack; MI—myocardial infarction; COPD—chronic obstructive pulmonary disease; CV—cardiovascular; RAAS—renin–angiotensin–aldosterone system; *p*—statistical significance according to the χ^2^ test; * statistical significance according to the Mann–Whitney U test; in the case of statistical significance (*p* < 0.05), the values are expressed in bold. ^a^ The time period covered by “history”—5 years before the index date of the analysis of reinitiation/analysis of discontinuation among reinitiators. ^b^ Antiplatelet agent—in the analysis of reinitiation, the last antiplatelet agent before discontinuation in our previous study [15]/in the analysis of discontinuation among reinitiators, the antiplatelet agent administered initially at the time of reinitiation. ^c^ Patient co-payment—calculated as the cost of antiplatelet treatment paid by the patient per month; in the analysis of reinitiation, co-payment for the last antiplatelet agent before discontinuation in our previous study [15]/in the analysis of discontinuation among reinitiators, co-payment for the antiplatelet agent administered initially at the time of reinitiation. ^d^ New antiplatelet agent user—patient in whom antiplatelet treatment was initiated in association with the diagnosis of peripheral arterial disease. ^e^ Lipid-lowering agents other than statins—ezetimibe and fibrates. ^f^ in the analysis of reinitiation, duration of persistence before discontinuation in our previous study [15]/in the analysis of discontinuation among reinitiators, duration of the period of nonpersistence (before reinitiation).

**Table 2:** Multivariate analysis of the association between patient- and medication-related characteristics and the likelihood of reinitiation/discontinuation among reinitiators.

Factor	Analysis of Reinitiation (*n* = 3032)	Analysis of Discontinuation Among Reinitiators (*n* = 2006)
	HR (95% CI)	HR (95% CI)
*Socio-demographic characteristics*		
Age	0.99 (0.98–1.00)	**0.98 (0.97–0.99)**
Female sex	1.05 (0.95–1.16)	1.11 (0.96–1.28)
University education	1.00 (0.85–1.19)	**1.41 (1.12–1.77)**
Employed patients	1.02 (0.84–1.23)	0.86 (0.66–1.12)
*History of cardiovascular events ^a^*		
History of ischemic stroke	**0.82 (0.72–0.93)**	**0.82 (0.70–0.96)**
History of TIA	1.06 (0.91–1.24)	0.95 (0.78–1.16)
History of MI	0.85 (0.70–1.03)	1.02 (0.81–1.29)
*Cardiovascular events during non-persistence/period of reinitiation*		
Ischemic stroke	**1.48 (1.21–1.81)**	0.74 (0.53–1.04)
TIA	1.29 (0.98–1.69)	1.23 (0.77–1.96)
MI	**1.63 (1.26–2.12)**	1.01 (0.64–1.59)
*Comorbid conditions*		
Number of comorbid conditions	0.95 (0.84–1.08)	1.12 (0.95–1.33)
Arterial hypertension	1.06 (0.89–1.27)	0.86 (0.67–1.10)
Chronic heart failure	1.02 (0.79–1.32)	1.26 (0.90–1.76)
Atrial fibrillation	**0.67 (0.54–0.84)**	0.90 (0.67–1.20)
Diabetes mellitus	0.97 (0.83–1.14)	**0.76 (0.62–0.94)**
Hypercholesterolemia	1.13 (0.96–1.33)	0.93 (0.75–1.16)
Dementia	0.98 (0.77–1.25)	0.89 (0.64–1.25)
Depression	0.99 (0.81–1.21)	0.94 (0.72–1.23)
Anxiety disorders	1.02 (0.86–1.20)	0.97 (0.78–1.21)
Parkinson’s disease	1.01 (0.77–1.33)	0.80 (0.54–1.18)
Epilepsy	1.34 (0.97–1.86)	0.79 (0.49–1.27)
Bronchial asthma/COPD	**1.21 (1.02–1.43)**	0.96 (0.76–1.22)
*Antiplatelet agent related characteristics*		
Antiplatelet agent ^b^		
Aspirin	1.00	1.00
Clopidogrel	**0.70 (0.61–0.81)**	1.04 (0.85–1.27)
Ticlopidine		1.39 (0.97–1.99)
Aspirin + Clopidogrel	**0.54 (0.37–0.79)**	0.67 (0.44–1.02)
Patient´s co-payment (EUR) ^c^	1.03 (0.97–1.10)	**0.90 (0.82–0.98)**
New antiplatelet agent user ^d^	0.93 (0.81–1.06)	1.12 (0.93–1.34)
General practitioner as index prescriber	1.03 (0.93–1.14)	0.96 (0.83–1.10)
*Cardiovascular co-medication*		
Number of medications	1.01 (0.99–1.04)	0.98 (0.95–1.01)
Number of CV medications	1.00 (0.95–1.05)	1.02 (0.96–1.09)
Anticoagulants	1.00 (0.87–1.13)	1.02 (0.86–1.21)
Cardiac glycosides	**0.67 (0.51–0.86)**	1.00 (0.70–1.43)
Antiarrhythmic agents	0.89 (0.72–1.11)	1.17 (0.87–1.56)
Beta-blockers	**0.84 (0.73–0.96)**	1.06 (0.88–1.28)
Thiazide diuretics	1.00 (0.88–1.13)	0.92 (0.78–1.10)
Loop diuretics	0.87 (0.75–1.01)	1.08 (0.88–1.33)
Mineralocorticoid receptor antagonists	0.85 (0.66–1.11)	0.77 (0.52–1.12)
Calcium channel blockers	1.11 (0.98–1.25)	0.87 (0.74–1.03)
RAAS inhibitors	0.90 (0.78–1.03)	1.12 (0.93–1.36)
Statin	1.07 (0.96–1.19)	0.92 (0.79–1.06)
Lipid lowering agents other than statins ^e^	0.94 (0.80–1.10)	0.96 (0.78–1.19)
Duration of persistence/non-persistence (months) ^f^	**0.99 (0.98–0.99)**	1.00 (0.99–1.01)

Values represent hazard ratios HR (95% confidence intervals). In the case of statistical significance (*p* < 0.05), the values are expressed in bold. TIA—transient ischemic attack; MI—myocardial infarction; COPD—chronic obstructive pulmonary disease; CV—cardiovascular; RAAS—renin–angiotensin–aldosterone system. ^a^ The time period covered by “history”—5 years before the index date of the analysis of reinitiation/analysis of discontinuation among reinitiators. ^b^ Antiplatelet agent—in the analysis of reinitiation, the last antiplatelet agent before discontinuation in our previous study [15]/in the analysis of discontinuation among reinitiators, the antiplatelet agent administered initially at the time of reinitiation. ^c^ Patient co-payment—calculated as the cost of antiplatelet treatment paid by the patient per month; in the analysis of reinitiation, co-payment for the last antiplatelet agent before discontinuation in our previous study [15]/in the analysis of discontinuation among reinitiators, co-payment for the antiplatelet agent administered initially at the time of reinitiation. ^d^ New antiplatelet agent user—patient in whom antiplatelet treatment was initiated in association with the diagnosis of peripheral arterial disease. ^e^ Lipid-lowering agents other than statins—ezetimibe and fibrates. ^f^ In the analysis of reinitiation, duration of persistence before discontinuation in our previous study [15]/in the analysis of discontinuation among reinitiators, duration of the period of nonpersistence (before reinitiation).

## Data Availability

The data that support the findings of this study are available from the General Health Insurance Company but restrictions apply to the availability of these data, which were used under license for the current study, and so are not publicly available. Data are however available from the authors upon reasonable request and with permission of the General Health Insurance Company.
